# Spermatogonial Stem Cell Markers and Niche in Equids

**DOI:** 10.1371/journal.pone.0044091

**Published:** 2012-08-28

**Authors:** Guilherme M. J. Costa, Gleide F. Avelar, José V. Rezende-Neto, Paulo Henrique A. Campos-Junior, Samyra M. S. N. Lacerda, Bruno S. C. Andrade, Ralph Gruppi Thomé, Marie-Claude Hofmann, Luiz R. Franca

**Affiliations:** 1 Laboratory of Cellular Biology, Department of Morphology, Federal University of Minas Gerais, Belo Horizonte, Minas Gerais, Brazil; 2 Unit 1105, Department of Endocrine Neoplasia and Hormonal Disorders, MD Anderson Cancer Center, University of Texas, Houston, Texas, United States of America; University Hospital of Münster, Germany

## Abstract

Spermatogonial stem cells (SSCs) are the foundation of spermatogenesis and are located in a highly dynamic microenvironment called “niche” that influences all aspects of stem cell function, including homing, self-renewal and differentiation. Several studies have recently identified specific proteins that regulate the fate of SSCs. These studies also aimed at identifying surface markers that would facilitate the isolation of these cells in different vertebrate species. The present study is the first to investigate SSC physiology and niche in stallions and to offer a comparative evaluation of undifferentiated type A spermatogonia (Aund) markers (GFRA1, PLZF and CSF1R) in three different domestic equid species (stallions, donkeys, and mules). Aund were first characterized according to their morphology and expression of the GFRA1 receptor. Our findings strongly suggest that in stallions these cells were preferentially located in the areas facing the interstitium, particularly those nearby blood vessels. This distribution is similar to what has been observed in other vertebrate species. In addition, all three Aund markers were expressed in the equid species evaluated in this study. These markers have been well characterized in other mammalian species, which suggests that the molecular mechanisms that maintain the niche and Aund/SSCs physiology are conserved among mammals. We hope that our findings will help future studies needing isolation and cryopreservation of equids SSCs. In addition, our data will be very useful for studies that aim at preserving the germplasm of valuable animals, and involve germ cell transplantation or xenografts of equids testis fragments/germ cells suspensions.

## Introduction

Spermatogonial stem cells (SSCs) are the undifferentiated male germ cells committed to the establishment and maintenance of spermatogenesis [1]. These cells are capable of self-renewal (providing a pool of A single or As spermatogonia) and differentiation, leading to the formation of Apaired (Apr), Aaligned (Aal) and differentiating spermatogonia (A1–4, In and B; in rodents) [1]–[5]. In horses and donkeys, it is already established that Aal spermatogonia differentiate into A1 spermatogonia that produce A2 and A3 spermatogonia, which give rise to type B1 and B2 spermatogonia [6], [7]. Recent studies in mice demonstrated that undifferentiated spermatogonia (As to Aal) maintain the stemness potential [8], where Apr and Aal cells are able to produce new As spermatogonia by spermatogonial clones fragmentation [8].

In addition to transmitting genetic information to the next generation, and being capable to repopulate the germ cell-depleted testis through the germ cell transplantation technique [9], [10], SSCs are also able to convert into pluripotent cells that differentiate into somatic tissues [11]. Therefore, investigating SSCs physiology is a crucial aspect of reproductive biology, leading to a better understanding of some causes of male infertility, to the development of novel reproductive biotechnologies [12] and to the generation of novel cellular models for tissue engineering [11], [13]. In this context, many studies have been developed aiming at identifying specific markers for these cells in vertebrates [14], [15]. Particularly, a specific SSC marker would be very helpful for the characterization and isolation of these cells [16]. This would facilitate the application of different biotechnologies aiming at preserving the germplasm [17], by using for instance the germ cell transplantation technique [10] or transdifferentiation approaches [11].

Three SSC markers involved in the regulation of self-renewal and themaintenance of the SSC pool in mice have gained special attention. One is a transcription factor known as PLZF (promyelocytic leukaemia zinc finger) and the others are membrane receptors named GFRA1 (GDNF family receptor alpha-1) and CSF1R (Colony stimulating factor 1 receptor) [1], [18]–[24]. Studies performed in the horse have demonstrated that subpopulations of spermatogonia (mainly As) present specific surface glycosylation pattern and this same population of cells are positively labeled for DBA (Lectin, *Dolichos biflorus* agglutinin) [16], [25], [26] and CT1 (carbohydrate-specific antibody) [16]. However, to date none of the markers that are specific for SSCs in mice and other species have been studied in equids.

As reviewed by Oatley and Brinster (2012) [1], in the testis a balance of SSCs self-renewal and differentiation must be tightly regulated to ensure continuous spermatogenesis. Signals emanating from a specific microenvironment called “niche” influence all aspects of stem cell function, including self-renewal, differentiation, and apoptosis. Therefore, defining the components of SSC niches in mammalian testes is important for understanding the foundation of sustained spermatogenesis [1]. By definition, the SSC niche is a rich microenvironment formed by growth factor contributions of somatic support cells, including Sertoli, Leydig, and peritubular myoid cells [1]. Recent evidence indicates that Sertoli cells play a major role in establishing the SSC niche in mouse testes, and they may achieve this through orchestrating the contributions of other somatic cell populations [1], particularly those located in the interstitial compartments as found for laboratory rodents [27]–[29] and donkeys [7]. In this regard, Yoshida and collaborators reported that the vascular network probably plays an important role in determining the SSC niche [30]. A recent study from our laboratory, which used the collared peccary as a model, suggested similar cell-vasculature interactions and also indicated that Leydig cells may induce SSCs to differentiate [31].

Few reports in the literature have investigated the biology of equid spermatogonia [6], [7], [16]. Therefore, the main objective of the present study was to perform a detailed and comprehensive morphofunctional evaluation of the horse testis. We specifically analyzed spermatogonial kinetics and molecular markers expressed by these cells to understand the SSC niche in this species. Further, we developed comparative studies for two other domestic equid species (donkey and mules). We also investigated the SSC niche during horse breeding and non-breeding seasons.

## Materials and Methods

### Ethics Statement

All surgical procedures were performed by a veterinarian and followed approved guidelines for the ethical treatment of animals. The protocol was approved by the Committee on Animal Experimentation-CETEA, Federal University of Minas Gerais (Permit Number: 056/11). All surgery was performed under anesthesia and analgesia, and all efforts were made to minimize suffering. The animals were anaesthetized with acepromazine and xylazine intravenously followed 15 minutes later by ketamine. Following xylazine administration, lidocaine was administered subcutaneously along the incision line and by intrafunicular injection. Prophylactic and postoperative antibiotic and anti-inflammatory therapy was performed using benzylpenicillin and phenylbutazone respectively.

### Animals and Tissue Preparation

Fourteen adult stallions (n = 14), two adult donkeys (n = 2) and four adult mules (n = 4) were used in this study. The animals were obtained from farms located in the Southeast region of Brazil (Minas Gerais province). For stallions, testes sampling was performed by orchiectomy during the breeding season (n = 7) (September to March in the South Hemisphere) and during the non-breeding season (n = 7). Testis from donkeys and mules were obtained during the breeding season.

After orchiectomy, the testes were separated from the epididymides and weighed, then cut longitudinally with a razor blade into small fragments. Testes from eight stallions were fixed by immersion in 4% buffered glutaraldehyde for 12 hours. Tissue samples measuring 2 to 3 mm in thickness were routinely processed and embedded in glycol methacrylate (Leica Historesin, Heidelberg, Germany) for histological and morphometric analyses. In order to characterize the different spermatogonial types using high resolution microscopy, samples measuring 1–2 mm in thickness were embedded in epoxy [27], [31]. As it will be described in more details in the western blotting analysis section, testes samples from stallions, mules and donkeys were stored at −80°C for Western blot analysis. Also, testis fragments from six stallions were fixed in Bouin’s solution for immunohistochemical evaluation.

### Spermatogonial Evaluation

#### Stages of the seminiferous epithelium cycle

Stages of the seminiferous epithelium cycle (SEC) were characterized in stallions based on the development of the acrosomic system, morphology of the developing spermatid nucleus and in the overall germ cells associations [7], [32], [33]. The relative stage frequencies were determined evaluating 150 seminiferous tubule cross sections per each animal, at 400× magnification.

#### Spermatogonial morphology, size and kinetics

We characterized the morphology of different spermatogonial types in stallions by analyzing images obtained from each stage of the SEC [27], [31]. For this purpose, the following morphological nuclear features were evaluated: nucleus shape, presence and disposition of heterochromatin, euchromatin granularity, and extent of nucleolus compaction [27], [31]. For each stage of the SEC, spermatogonia were grouped according to their morphological characteristics. The nuclear volume of each spermatogonial type was obtained by measuring the diameter of 30 nuclei of each cell type per animal [31]. Spermatogonial kinetics was performed by counting the different spermatogonial types present in each stage of the SEC and their number were expressed as a ratio per 1000 Sertoli cell nuclei [7], [31]. Spermatogonial morphology for donkeys and mules was similar to that described for stallions, and followed the criteria previously described in our laboratory [7], [34].

### Western Blotting and Immunostaining Analyses

#### Western blotting analysis

To qualitatively evaluate the presence of specific markers for SSCs (GFRA1, PLZF and CSF1R), immunoblots were performed using total protein lysates from stallion (during the breeding and non-breeding seasons), donkey and mule testes stored at −80°C. For this evaluation, 300 mg of testis parenchyma from each individual investigated were placed in 0.9% NaCl containing protease inhibitors (Sigma Aldrich’s Corp., St. Louis, MO, USA), and the tissues were homogenized and sonicated. After that, the lysates were centrifuged at 14,000×g for 20 minutes. Supernatants were collected, and then frozen at −80°C. Protein samples were diluted to 1∶2 in a solution of 10% sodium dodecyl sulfate (SDS, Sigma Aldrich), 2% glycerol, 0.2% bromophenol blue and, when reducing conditions were required, 1% beta-mercaptoethanol was added in 0.5 M TRIS buffer pH 6.8. The samples were boiled for 5 minutes. Denaturing 12% SDS polyacrylamide mini-gels were prepared and 25 µL samples were loaded into the wells. High-molecular-weight markers (Sigma Aldrich) were run parallel to the samples. Gels were run under a 90 mA current and the separated proteins were transblotted to nitrocellulose membrane for 75 minutes using a 100 mA current. The strips were blocked with 3% BSA (Sigma Aldrich) in TBS for 1 hour at room temperature. Proteins were detected through 90 minutes incubation, at room temperature, with one of the following primary antibodies diluted in TBS 1% BSA: polyclonal goat anti-GFRA1 (1∶500, SC-6157, Santa Cruz Biotechnology), monoclonal mouse anti-PLZF (1∶200, OP128, Calbiochem) and polyclonal rabbit anti-CSF1R (1∶200, ab61137, Abcam). Subsequently, the strips were rinsed three times with TBS 0.05% Triton-X buffer and then incubated for 1 hour with biotinylated anti-goat (for GFRA1; 1∶100, ab6740, Abcam), anti-mouse (for PLZF; 1∶500, SAB4600004, Sigma) or anti-rabbit (for CSF1R; 1∶200, ab6720, Abcam) IgG antibodies at room temperature. Before exposing the strips to streptavidin solution (Thermo Scientific, TS-125-HR) for 15 minutes at room temperature, they were rinsed three times with TBS 0.05% Triton-X buffer. Prior developing the reaction, the samples were then rinsed twice with TBS 0.05% Triton-X and once with pure TBS buffer. The strips were exposed to DAB solution (Sigma Aldrich) in TBS containing chloronaphthol (Sigma Aldrich), methanol and hydrogen peroxide (Sigma Aldrich) during approximately 1 minute and then washed in water. Finally, the strips were scanned with an Epson Perfection 4990 photo scanner.

#### Immunostaining analyses

In order to evaluate the *in situ* expression of proteins analyzed by Western blotting, we performed immunostaining using the immunoperoxidase method. Slides were analyzed by light microscopy. The tissue samples were fixed in Bouin and embedded in paraplast (Sigma Aldrich). Five micrometer sections were immunostained using protocols specifically developed for each antigen and with antibody dilutions previously tested. After dewaxing and rehydration, antigen retrieval was performed in citrate buffer (pH 6.0) for 5 minutes after boiling in a microwave oven (total period of approximately 10 minutes). For immunohistochemistry, endogenous peroxidase was quenched for 30 minutes with 3% H_2_O_2_ (Sigma Aldrich) in PBS at room temperature. Non-specific binding was blocked with Ultra-V-Block (Thermo Scientific) for 30 minutes at room temperature. Primary antibodies GFRA1 (goat polyclonal, 1∶500, SC-6157, Santa Cruz Biotechnology), PLZF (mouse monoclonal, 1∶200, OP128, Calbiochem), CSF1R (rabbit polyclonal, 1∶200, ab61137, Abcam) and Cleaved Caspase-3 (rabbit polyclonal, 1∶300, Asp175, Cell Signaling Technology) were applied and the slides were incubated overnight at 4°C. The slides were exposed to biotinylated rabbit anti-goat (for GFRA1; ab 6740, 1∶100, Abcam), horse anti-mouse (for PLZF; pk6102, 1∶1000, VectorStain ABC kit, Vector Laboratories) and goat anti-rabbit (for CSF1R and Caspase-3; ab6720, 1∶200, Abcam) immunoglobulin G (IgG) antibodies during 60 minutes at room temperature. Detection of the signal was performed by incubating the sections in streptavidin for 10 minutes at room temperature, followed by the reaction with peroxidase substrate 3,3′-diaminobenzidine (DAB, Sigma Aldrich) and counterstaining with hematoxylin (Merck). Following dehydration, sections were mounted and analyzed.

In order to quantify the percentage of GFRA1(+), PLZF(+) and CSF1R(+) in type A undifferentiated spermatogonia (Aund) (previously characterized according to morphological criteria), 200 Aund cells were randomly evaluated for each stallion (n = 6), donkey (n = 2) and mule (n = 4). Only for stallions, qualitative double staining immunofluorescence was also performed in the present study. Non-specific background was blocked for 30 minutes with Ultra-V-Block (Thermo scientific) incubation. The proteins were detected using primary antibodies at the following dilutions: GFRA1 (1∶50), PLZF (1∶50) or CSF1R (1∶100). After the incubation with Alexa Fluor secondary antibodies [for GFRA1, 546 anti-goat (1∶500) and/or for PLZF and CSF1R, 488 anti-mouse (1∶500)] for 1 hour at room temperature, the confocal images were obtained using 40X and 60X oil immersion objectives in a 510 META Laser Scanning Confocal Microscope (Zeiss, Oberkochen, Germany), equipped with 488 and 543 nm lasers. Dual channel images were obtained by sequential scanning.

### Spermatogonial Distribution

#### Testis morphometry

The volume densities of the testicular tissue components in stallions were determined by images captured by light microscopy, using a 540-intersection grid from ImageJ software (National Institutes of Health, http://rsb.info.nih.gov/ij/). Fifteen randomly chosen fields/images (8100 points) were scored per testis for each animal (n = 8) at 200× magnification. In order to investigate the influence of seasonality in the testicular components, these stallions were divided separated in two groups of four animals.

#### Aund distribution in the seminiferous tubules

In order to investigate the distribution of Aund in stallions, both morphological and immunostaining (GFRA1+) evaluations were considered. In this regard, images from 10 seminiferous tubules cross-sections of each stage of the SEC previously characterized were obtained for each animal and the seminiferous tubules cross-sections were subdivided into four regions, as follows: I) adjacent to another tubule (Tubule-Tubule contact or TT); II) adjacent to the interstitial compartment without blood vessels (Tubule-Interstitium without blood vessels or TI−BV); III) adjacent to the interstitial compartment containing blood vessels (Tubule-Interstitium with blood vessels or TI+BV); and IV) adjacent to the interstitial compartment containing connective tissue (TIC). Considering that the tubular circumference corresponds to 360 degrees, the numbers of Aund cells obtained in the four different regions evaluated were expressed per degree [31]. Therefore, this approach allowed an estimation of the number of these cells located in each region. Also, to verify the possible effects of seasonality in the Aund cells distribution, this analysis was performed in testis samples collected during the breeding and non-breeding season. In order to verify the distribution of these cells facing the intertubular and tubular areas, the number of Aund was also expressed as the total number of cells per degree considering only two regions: TI (i.e. TI−BV + TI+BV + TIC); and TT.

#### Statistical analyses

Parametric data were analyzed by ANOVA and differences were compared by Tukey’s test, whereas the Student’s “t” test was used for two-parameter analysis. Non-parametric data were compared by Chi-square test. All analyses were performed using the GraphPad Prism 5 (GraphPad software, Inc). All data were expressed as mean ± SEM (standard error of the mean) and the significance level considered was p<0.05.

## Results

### Stages of the Seminiferous Epithelium Cycle

**Figure 1:**
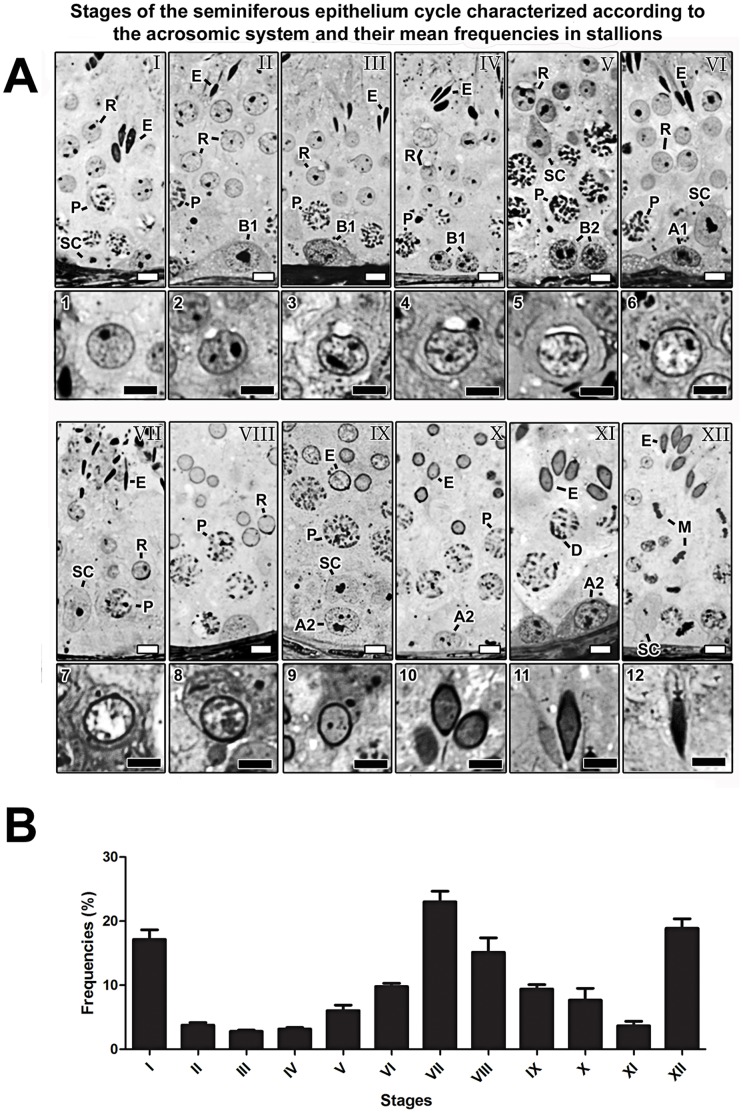
Stages of the seminiferous epithelium cycle (A) and their frequencies (B) in stallions. A) The following symbols were used to designate specific germ cell types: A1, type A1 spermatogonia; A2, type A2 spermatogonia; B1, type B1 spermatogonia; B2, type B2 spermatogonia; P, pachytene spermatocyte; D, diplotene spermatocyte; M, meiotic figure; R, round spermatids; E, elongating/elongated spermatids; SC, Sertoli cell. Arabic numerals (1–12) indicate each step of the spermatid acrosome development. B) Note that stages I, VII and XII presented the highest frequencies, whereas the opposite was observed for stages II, III, IV and XI. White and black bars = 5 µm.

**Figure 2:**
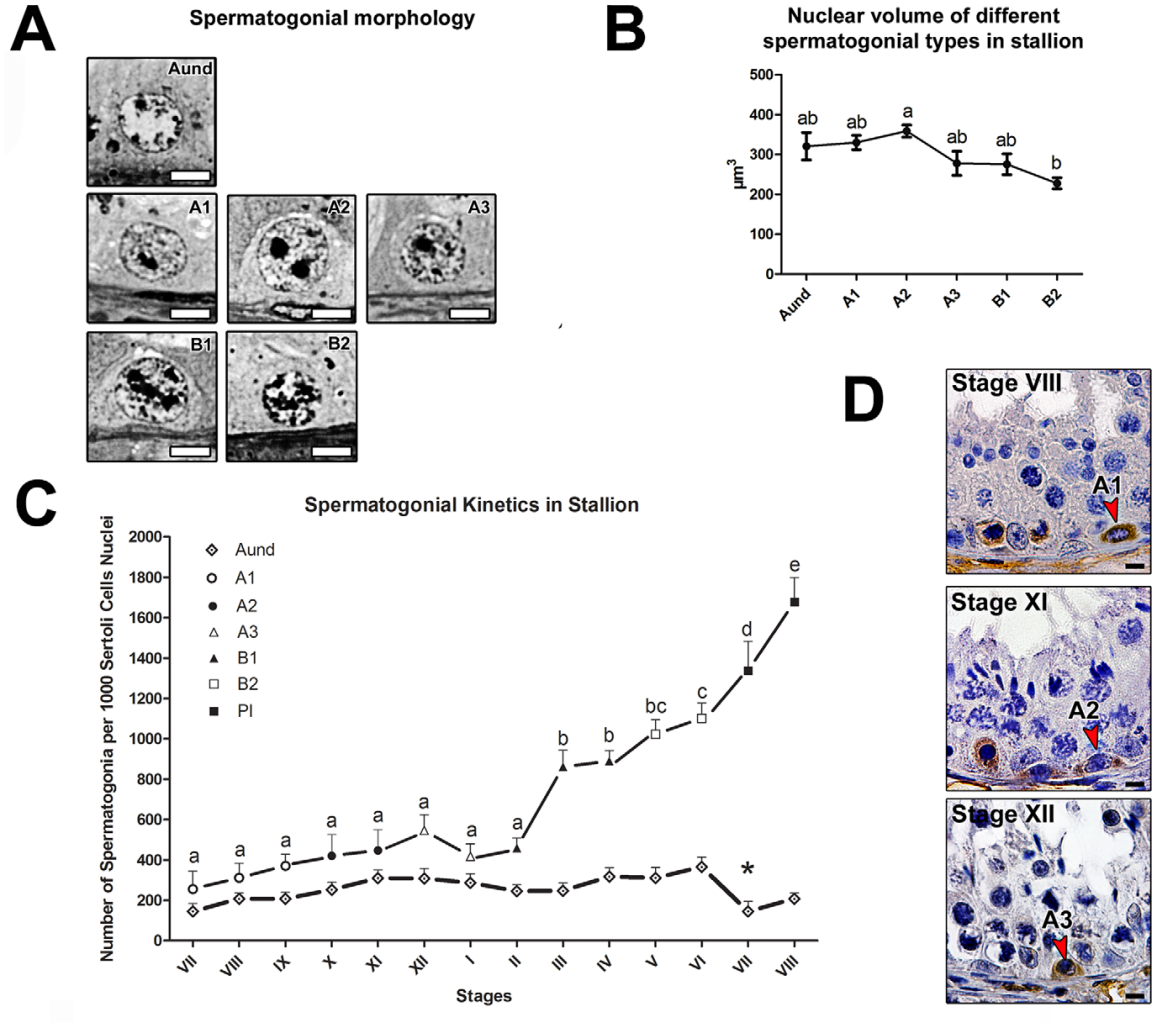
Spermatogonial types and their kinetics in the stallion seminiferous epithelium cycle. A) High-resolution light photomicrographies of spermatogonial cells: Aund and differentiated spermatogonia (type A1, A2, A3, B1 and B2), showing their nuclear size and details that allowed their morphological identification. B) Nuclear volume of the different spermatogonial types characterized showing that A2 presented the highest value, particularly in comparison to type B2 spermatogonia. C) Number (kinetics) of Aund and differentiated spermatogonial cells and preleptotene spermatocytes (Pl) per 1000 Sertoli cell nuclei. Note that, except for stages I and II, the values obtained for differentiated spermatogonia increased gradually, whereas the numbers of Aund were relatively stable, reaching their lowest level at stage VII. D) Illustration of the immunolocalization of active caspase-3 in differentiated type A (A1, A2, and A3) spermatogonial cells. Figures A and D, bar = 5 µm.

Based on the development of the acrosomal system in spermatids, twelve stages of the SEC were characterized in stallions (Figure 1A; I–XII). The inserts containing arabic numbers (1 to 12) below each stage in this figure depict the developmental steps of the acrosomic system in spermatids. This is briefly described as follows: the spermiogenic phase started with step 1 spermatids in which acrosomal vesicles cannot be visualized (Figure 1A; 1). Subsequently, a granular vesicle developed (Figure 1A; 2), which gradually flattened and spread laterally over the nucleus surface of round spermatids (Figure 1A; 3–5). In the following stages (Figure 1A; 6–7), the angle formed by the acrosome at the nucleus surface increased progressively, reaching its maximal size at stage VIII (Figure 1A; 8). From this stage on, spermatids nuclei progressively elongated and acrosomes became oriented towards the base of the seminiferous tubules (Figure 1A; 9–12). Spermiation ocurred at stage VII. Mean frequence percentages of each of the twelve stages characterized for stallion are shown in Figure 1B. Stages I, VII, and XII presented the highest frequencies, while the lowest frequencies were observed for stages II, III, IV, and XI.

### Spermatogonial Morphology, Size and Kinetics

Based on the criteria previously described in our laboratory for donkeys [7] and peccaries [31], spermatogonial cells in stallions were characterized as type A undifferentiated (Aund) and differentiated (A1, A2, A3) and type B (B1 and B2) (Figure 2A). Due to the absence of clear morphological criteria, Aund spermatogonial cells encompassed A single (As), A paired (Apr), and A aligned (Aal). Morphologically, the Aund spermatogonium showed a mottled or granular spherical nucleus with little heterochromatin. In overall, A1, A2 and A3 spermatogonia presented a light and finely granular euchromatin and their heterochromatin gradually increased from A1 to A3. Type B1 and B2 spermatogonia showed ovoid nuclei and presented an increased heterochromatin granularity, which gave to the nucleoplasm a very granular aspect. Morphometric analysis indicated that A2 spermatogonia exhibited the biggest nuclear volume, particularly when compared to type B2 spermatogonia (p<0.05; Figure 2B).

Contrary to Aund cells, which were present in all stages, differentiating spermatogonia were only observed in well-defined stages of the SEC (Figure 2C). In this regard, type A1 cells were found at stages VII, VIII and IX, type A2 cells were observed at stages X and XI, while type A3 cells were present at stages XII and I. With reference to type B spermatogonia, B1 cells were observed at stages II, III and IV, while B2 cells were found at stages V and VI. The kinetics of differentiating spermatogonia indicated a gradual increase in cell numbers from type A1 to type B2 spermatogonia, with a decrease from A3 to B1 that was however not significant. As illustrated in Figure 2D, caspase-3 immunostaining frequently detected apoptosis in differentiating type A spermatogonia, which explains that the increase in cell number was not exponential. With reference to Aund spermatogonia (Figure 2C), our kinetics studies indicated that these cells proliferated, with the highest cell number observed at stage VI, just before they were expected to differentiate into A1 cells. Subsequently, an abrupt decrease (p<0.05) was observed at stage VII, while their number increased again throughout the following stages. Overall however, Aund cell numbers were not significantly different throughout the stages of spermatogenesis, except for stage VII.

### GFRA1, PLZF and CSF1R Immunostaining and Western Blotting

**Figure 3:**
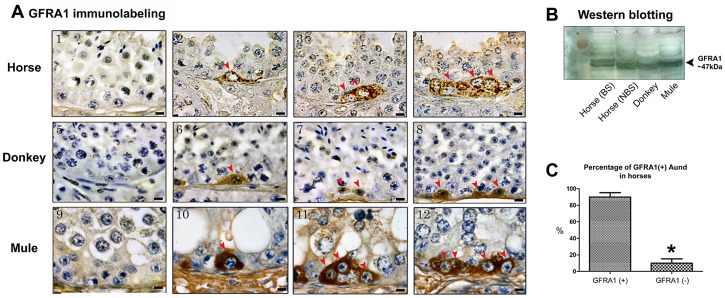
Immunostaining evaluation of the presence of GFRA1 in equids. A) As it can be noted, the expression of this marker was limited to the cytoplasm of Aund (arrowheads) and this pattern was similar for horse (A2–4), donkey (A6–8) and mule (A10–12). A1, A5 and A9 are the negative controls. B) Immunoblotting confirmed the expression of GFRA1 in the testis of horse [during the breeding (BS) and non-breeding (NBS) season], donkey and mule. C) Percentage of GFRA1(+) Aund cells showing that approximately 90% of these cells express this membrane receptor (*p<0.05). Figure A, bar = 10 µm.

**Figure 4:**
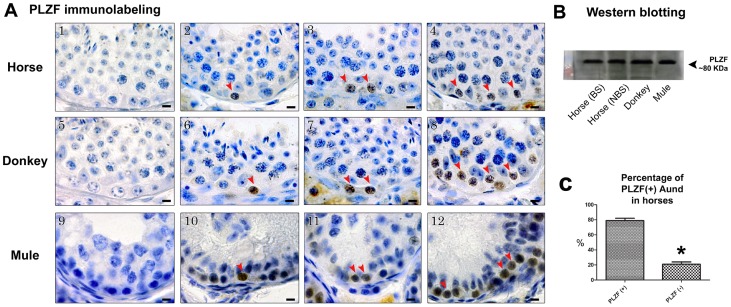
Immunostaining evaluation of the presence of PLZF in equids. A) As it can be observed, the expression of this marker was present in the nucleus of Aund (arrowheads) and this pattern was similar for the three equid species investigated (horse, A2–4; donkey, A6–8; and mule, A10–12). Negative controls are shown in A1, A5 and A9. B) Expression of PLZF was confirmed by immunoblotting in the horse testis [during the breeding (BS) and non-breeding (NBS) season], donkey and mule. C) Approximately 80% (*p<0.05) of Aund express this transcription factor. Figure A, bar = 10 µm.

**Figure 5:**
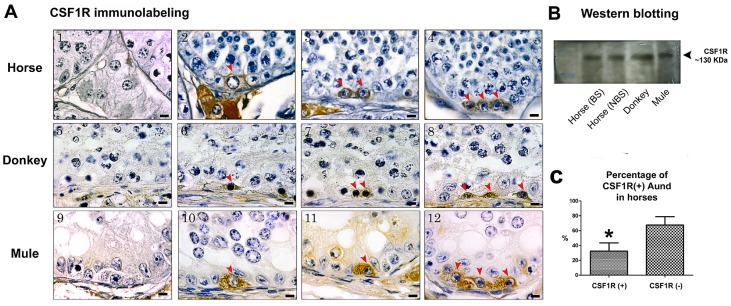
Immunostaining evaluation of the presence of CSF1R in equids. A) As it can be noted, the expression of this marker was limited to the cytoplasm of Aund (arrowheads) and this pattern was similar for horse (A2–4), donkey (A6–8) and mule (A10–12). A1, A5 and A9 are the negative controls. B) Immunoblotting confirmed the expression of CSF1R in the testis of horse [during the breeding (BS) and non-breeding (NBS) season], donkey and mule. C) Percentage of CSF1R(+) Aund cells showing that approximately 35% of these cells express this membrane receptor (*p<0.05). Figure A, bar = 10 µm.

Using immunohistochemistry, we detected expression of GFRA1, PLZF and CSF1R by Aund spermatogonia in all three equid species investigated. Aund cells could be visualized as single, pairs and chains of spermatogonia (Figures 3A, 4A, and 5A). While expression of the transcription factor PLZF was limited to the nucleus of Aund (Figure 4A; arrowheads), expression of both membrane receptors GFRA1 and CSF1R was confined to the membrane/cytoplasm of these cells (Figures 3A and 5A; arrowheads). Expression of GFRA1 (∼47 KDa), PLZF (∼80 KDa), and CSF1R (∼130 KDa) was confirmed by immunoblotting of testis lysates obtained from horses (during the breeding and non-breeding season), donkeys and mule (Figures 3B, 4B, 5B).

### Percentage of Aund Cells Positive for GFRA1, PLZF and CSF1R

**Figure 6:**
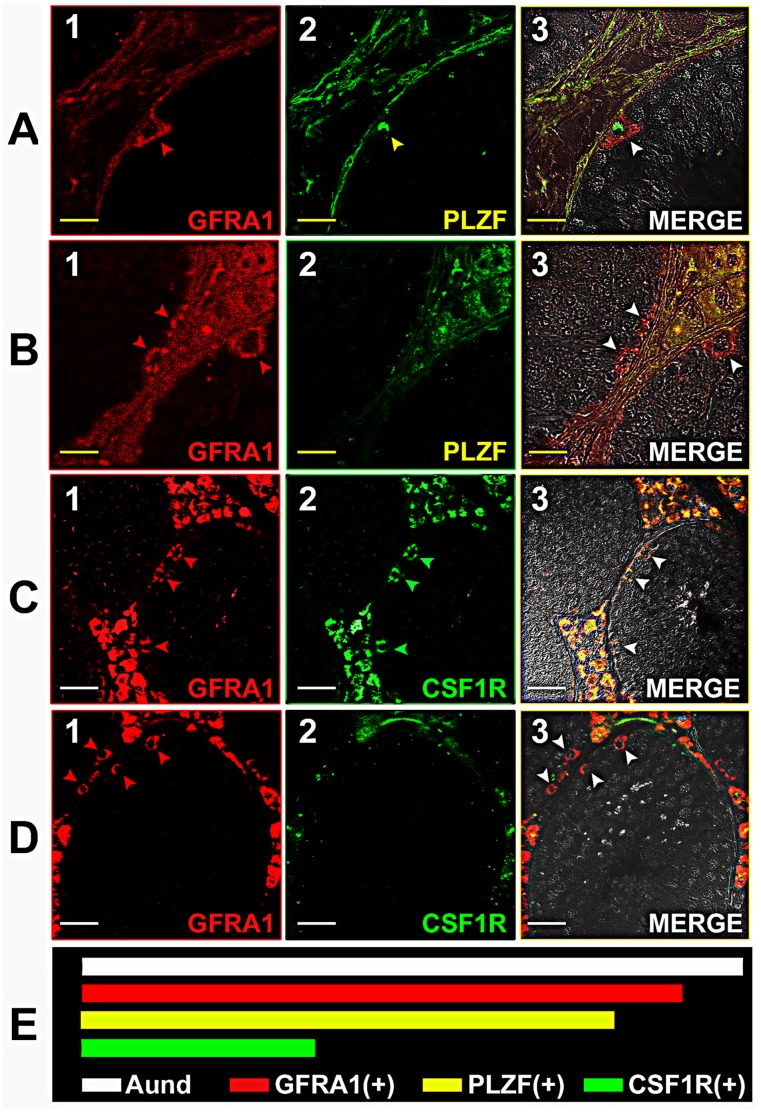
Qualitative evaluation of the co-localization of the three different spermatogonial markers used for horses. Considering the co-expression of GFRA1 and PLZF the following pattern was observed: A) GFRA1(+) cells (A1; red arrowhead) presenting co-localization with PLZF (A2; yellow arrowhead), as evidenced in the merged figure (A3; white arrowhead); B) this panel illustrates GFRA1(+) cells (B1; red arrowhead) that do not present PLZF expression (B2), as shown in the merged figure (B3; white arrowhead). In relation to the co-expression of GFRA1 and CSF1R the following labeling pattern was observed: C) GFRA1(+) cells (C1; red arrowhead) also expressing CSF1R (C2; green arrowhead), shown in the merged figure (C3; white arrowhead); D) differently, some GFRA1(+) cells (D1; red arrowhead) do not present CSF1R (D2; white arrowhead in D3 merged figure). E) Summarization of the quantitative data obtained for Aund GFRA1, PLZF and CSF1R positive cells in horses, suggesting that these three proteins are differently expressed in this cell population. Yellow bar = 20 µm; White bar = 30 µm.

As explained above, Aund include A single (As), A paired (Apr), and A aligned (Aal) spermatogonia. In the immunolabeling quantitative analysis for horses, we observed that approximately 90 and 80% of Aund cells expressed GFRA1 and PLZF respectively (Figures 3C and 4C). In contrast, only one third of these cells were labeled for CSF1R (Figure 5C). In order to better understand marker distribution, a qualitative double-labeling analysis was performed for GFRA1, PLZF, and CSF1R (Figure 6). Our data suggest that these proteins are differently expressed in distinct subsets, or subpopulations, of Aund spermatogonia. Indeed, some GFRA1 positive cells were negative for PLZF or CSF1R (Figures 6B and 6D). Figure 6E summarizes the percentages of Aund immunolabeled for the three markers evaluated in horses. The results obtained for the immunolabeling quantitative analysis performed for donkeys (D) and mules (M) were very similar to those obtained for horses, as follow: GFRA1 (D: 87.5±2.5%; M: 93±1%); PLZF (D: 76.5±3.5%; M: 83.5±1.5%); and CSF1R (D: 26.5±5.5%; M: 35.5±2.5%).

### Testicular Morphometric Analysis

**Figure 7:**
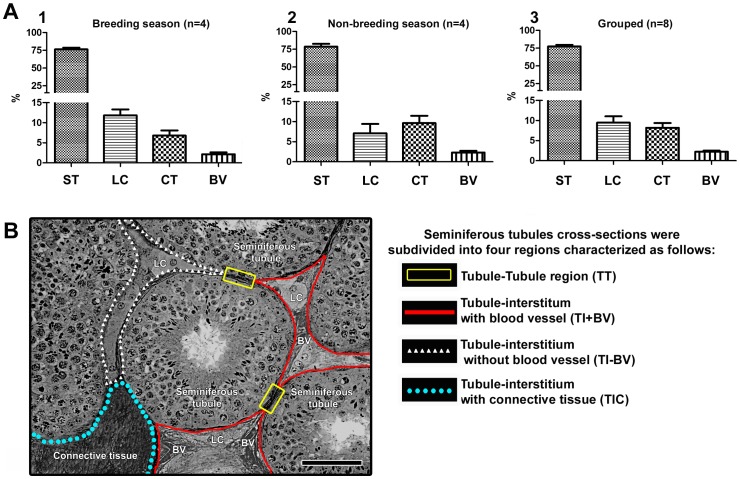
Testis morphometry in horses, during the breeding and non-breeding season, and seminiferous tubules cross-sections subdivisions. A) Whereas the seminiferous tubules (ST) volume density was not changed during the two periods evaluated, Leydig cells (LC) and connective tissue (CT) were the most prevalent components of the intertubular compartment during the breeding and non-breeding season. Seminiferous tubules cross-sections were subdivided into 4 different regions according to the prevalence of these aforementioned components (B). BV = blood vessels. Figure B, bar = 100 µm.

We measured the volume density of components of the testicular parenchyma in stallions during the breeding (Figure 7A; 1) and non-breeding seasons (Figure 7A; 2). In both periods evaluated, we observed that the seminiferous tubules occupied about 75% of the testis parenchyma. During the breeding season, half of the intertubular compartment was occupied by Leydig cells, while in the non-breeding period connective tissue was the predominant component (∼40%) of this compartment. Connective tissue occupied approximately one third of the interstitium in the breeding season. The volume density of blood vessels was very similar in the two periods investigated. Based on the percentage of testicular components in the testis parenchyma, and in order to investigate spermatogonial distribution, cross-sections of horses seminiferous tubules were subdivided into 4 different regions (Figure 7B): I) adjacent to another tubule (TT); II) next to the interstitial compartment with blood vessels (TI+BV); III) adjacent to the interstitial compartment without blood vessels (TI-BV); and IV) next to the interstitial compartment containing connective tissue (TIC).

### Spermatogonial Distribution

**Figure 8:**
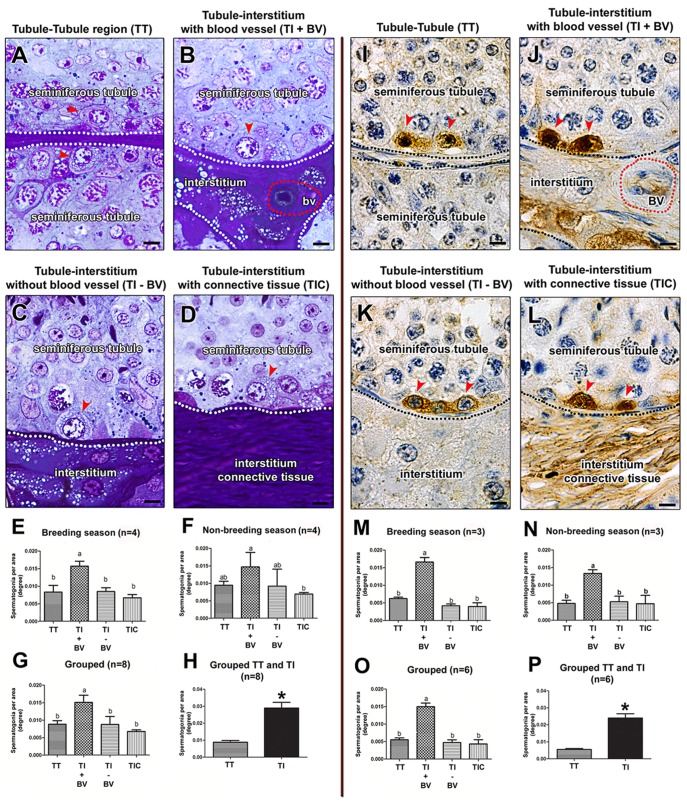
Aund distribution in horses according to morphological (A–H) and immunostaining (I–P) criteria. As indicated by red arrowheads, using both criteria, Aund cells were present in all four regions considered. However, independently of the breeding season, these cells were more frequently observed in the areas facing the interstitium, particularly nearby the blood vessels. TT = Tubule-Tubule contact; TI−BV = Tubule-Interstitium without blood vessels; TI+BV = Tubule-Interstitium with blood vessels; TIC = Tubule-Interstitium containing connective tissue. Figure A–D and H–K, bar = 10 µm.

According to the four seminiferous tubules regions considered, and based on morphological and GFRA1 immunolabeling evaluations (Figure 8A–P), GFRA1(+) Aund spermatogonia were predominantly located (p<0.05) in the areas where the tubule faced the interstitial compartment containing blood vessels (TI+BV) (Figures 8E–G and 8M–O). Except for the blood vessels area, GFRA1(+) Aund spermatogonia distribution was similar in the other three areas investigated. As clearly evidenced in Figure 8H and P, the total number of Aund facing the interstitium is about 3-fold higher than the total number of Aund located in the tubule-tubule area (p<0.05). Also, using both morphological and immunostaining evaluations, Aund distribution pattern was not affected by seasonality.

## Discussion

To our knowledge, the present study in the first that investigates the SSC niche in stallions. This study also offers a comparative evaluation of Aund markers in three different domestic equid species (stallions, donkeys and mules). In order to better understand the topology of cellular compartments within the testis, we first characterized the stages of the SEC in stallions according to acrosome development in spermatids. We also characterized the different types of spermatogonia in this species and investigated their distribution in four different regions of seminiferous tubules cross-sections, using morphological and Aund markers criteria. Based on the distribution of GFRA1-positive spermatogonia, our findings strongly suggest that, similar to the other vertebrate species investigated in this respect, Aund cells in stallions were preferentially located in the areas facing the interstitium, particularly those nearby blood vessels. Also, all three Aund markers investigated (GFRA1, PLZF, and CSF1R) were expressed in stallions, donkeys and mules, which was confirmed by Western blot analysis. These results suggest that the molecular mechanisms driving SSCs physiology and maintenance of the niche are conserved among mammals.

The GFRA1 membrane receptor is considered one of the most useful markers of Aund [23]. Its presence at the surface of these germ cells has been described in mice [23], [35]–[38], pigs [39], domestic cats [40], [41], peccaries [31], non-human primates [42], [43], [44], [45] and humans [45], [46], [47]. The present study is the first to describe the expression of GFRA1 by Aund in equids. We believe that like in mice [18]–[20], [23], GFRA1 is important for SSCs self-renewal in equids. Quantification analysis, qualitatively confirmed by Western blotting, revealed a high prevalence of Aund cells labeled for GFRA1 (∼90%) in the stallion testis, suggesting that all subtypes of Aund cells (As, Apr and Aal) were expressing this receptor. The percentage of GFRA1 positive Aund cells found in the present work is similar to the values found for peccaries (∼93%) [31] and mice (90%) [48]. Because A single spermatogonia (As) represent approximately 10% of all Aund cells [5], [49], we believe that all Aund subtypes were labeled for GFRA1 in the equid species investigated in this study. However, in order to ascertain that all Aund subtypes express GFRA1, other studies will be necessary, such as whole-mount analysis [50]. In addition, further investigations will be needed to evaluate possible changes in GDNF (GFRA1 ligand) expression by Sertoli cells through the stages of the seminiferous epithelium in horses, as observed for rats [51] and mice [50].

Although slightly less frequent, expression of the transcription factor PLZF was observed in ∼80% of Aund in equids. This result, also confirmed by Western blotting, is similar to findings already described in rabbits [52]. PLZF is also highly conserved in mammals, and considered to be involved in self-renewal and maintenance of the SSC pool in mice [21], [22], sheep [53], rabbits and primates [42], [43], [52]. Based on these findings, we can assume that PLZF is also required for Aund/stem cell physiology in equids. In mice SSCs, PLZF activity represses *Kit* and *Crtc1* gene expression, whose products are both involved in spermatogonial differentiation [54], [55]. Consequently, PLZF is co-expressed with OCT4, a marker for primitive stem cells. Future investigations should address these questions in equids.

In the present study, we also show that like in mice [24], [56] only one-third of Aund expressed the receptor for colony stimulating factor 1 (CSF1R). CSF1R expression was also confirmed by Western blotting. In the collared peccary, double labeling demonstrated that Aund spermatogonia positive for CSF1R also expressed GFRA1 [31]. Other studies reported the presence of CSF1R in enriched mouse germ cell populations positive for THY1 [24] and GFRA1 [56]. Because colony stimulating factor 1 (CSF1) is secreted by Leydig and peritubular myoid cells in mice [24] and peccaries [31], it would be interesting to investigate a possible role of these somatic cells in the regulation of Aund in equids.

In comparison to the data obtained for rodents [21]–[24], [35]–[38], [48], [56], peccaries [31], and equids in the present study, and based on pluripotent markers (LIN28 and POU5F1) [57], the spermatogonial phenotype in man [52], [58] and in non-human primates [57], [58] seems to be rather different. Also, based on recent data showing that a great number of cytokines and growth factors, such as GDNF and CSF1, are produced by peritubular myoid cell, this somatic cell is now emerging as an important regulator of SSCs self-renew and niche [31], [46], [59]. Therefore, future studies should also address this important functional role of the peritubular myoid cells in equids.

To our knowledge, the present study is the first to investigate the distribution of GFRA1 positive spermatogonia in equids. Our data strongly suggest that the interstitium, particularly the vascular network, may play an important role in the regulation of equid SSCs, like in other species [30], [31]. Indeed, our findings demonstrate that stallion Aund spermatogonia positive for GFRA1 were preferentially located in the seminiferous tubules areas adjacent to blood vessels. Because there is yet no proven niche factor arising from the vasculature, this important feature is only an indirect reference point for the localization of the niche. However, it has been suggested that the concentration of follicle-stimulating hormone (FSH) is higher near blood vessels and this could stimulate GDNF (the GFRA1 ligand) production by Sertoli cells, creating a specific microenvironment for Aund [60], [61].

Due to drastic alterations in the intertubular compartment volume density during the sexually inactive period, an evident niche was not observed in golden hamsters [29]. In the present study, we did not observe any difference in the pattern of Aund distribution between breeding and non-breeding periods. These findings might be related to the fact that in countries where the annual photoperiod variation is less than two hours, effects of seasonality in stallions are not sufficient to promote significant changes in the testis interstitium composition [62]. In studies developed in Texas (USA), the total number of type A spermatogonia (undifferentiated plus differentiated) was significantly reduced in stallions during the non-breeding period [63], [64].

In the present investigation, we defined twelve stages of spermatogenesis in equids according to overall germ cells composition and acrosome development. A similar situation was previously described in donkeys [7]. This system of characterization is different from the so-called tubular morphology system, where eight stages of the SEC are always defined, including for stallions [65], [66], [67]. In addition, we obtained stages frequencies similar to the ones found for donkeys [7], even after grouping of the pre- and postmeiotic stages. This finding reinforces the hypothesis that stage frequencies may be phylogenetically determined among members of a same family [32], [68].

The different types of stallion spermatogonia exhibited morphological characteristics already described in donkeys [7]. Interestingly, the pattern found for nuclear volume, where the maximum value was observed for type A2 spermatogonia, was very similar to the one observed in mice [69]. As expected from the literature [5], [7], a gradual increase in cell numbers from type A1 to type B2 spermatogonia was observed in stallions. However, probably due to the high incidence of apoptosis or check points known to occur in the mammalian seminiferous epithelium [5], [70], we observed a significant germ cell loss, particularly in the transition from type A to type B spermatogonia [7]. Kinetics of Aund spermatogonia followed the trend observed in rodents [5], donkeys [7] and peccaries [31]. Indeed, the lowest germ cell number was observed at stage VII of the SEC where sperm release occurs and most of Aund become type A1 spermatogonia [5], [7], [31].

In summary, our findings suggest that the molecular mechanisms driving SSCs physiology and niche maintenance are conserved among mammals. Also, the characterization of Aund spermatogonia performed in the present study provides for the first time a foundation for future studies of SSCs self-renewal and differentiation in equids. Our data will facilitate isolation and cryopreservation of these cells. In addition, our findings will be very useful for studies involving germ cells transplantation and xenografts of equid testis fragments/germ cells suspensions to preserve the germplasm of valuable animals.
